# Commentary: Transdiagnostic Effects of Ventromedial Prefrontal Cortex Transcranial Magnetic Stimulation on Cue Reactivity

**DOI:** 10.3389/fnins.2018.00871

**Published:** 2018-11-23

**Authors:** Di Zhao, Mingming Zhang, Wenbo Luo, Tifei Yuan

**Affiliations:** ^1^Shanghai Key Laboratory of Psychotic Disorders, Shanghai Mental Health Center, Shanghai Jiao Tong University School of Medicine, Shanghai, China; ^2^Research Center of Brain and Cognitive Neuroscience, Liaoning Normal University, Dalian, China; ^3^Co-innovation Center of Neuroregeneration, Nantong University, Nantong, China

**Keywords:** transcranial magnetic stimulation, noninvasive brain stimulation, addiction, cue reactivity, ventromedial prefrontal cortex, function connectivity

The addictive brain undergoes dramatic changes as a result of drug exposure. A growing body of evidence suggests a wide range of brain areas, including ventromedial prefrontal cortex (VMPFC), orbitofrontal cortex (OFC), dorsolateral prefrontal cortex (DLPFC) and other subcortical regions, namely anterior cingulate cortex (ACC), striatum, and anterior insula, are involved in drug-related cue processing (for reviews, see Goldstein and Volkow, 2011 or Koob and Volkow, 2010; Dong et al., 2017; Egervari et al., 2018). Among them, VMPFC has been implicated as a core region across a spectrum of addictive disorder (Potenza et al., 2003; Moeller and Goldstein, 2014). Anatomical and functional connections (FC) between the VMPFC and limbic reward-related brain areas (e.g., nucleus accumbens, ACC, hippocampus and insula) make it a prominent candidate for bottom-up drug-related cue processing, self-related information evaluation and approachable/avoidable behavior modulation (Bzdok et al., 2013; Seo and Sinha, 2014). Research using functional magnetic image (fMRI) has identified dysfunction in inhibitory control mediated by VMPFC and limbic neural circuit in addicts compared to healthy controls (Bonson et al., 2002; Seo et al., 2013; Morein-Zamir and Robbins, 2015). Recent preclinical studies have implied that repetitive transcranial magnetic (rTMS) over the VMPFC can attenuate drug craving (Hanlon et al., 2015), which may be associated with neural activity in frontal-limbic brain areas in drug dependents (Hanlon et al., 2017a). However, the causal role of VMPFC in drug-related cue reactivity is still poorly understood. Also, it remains pivotal to dissect the TMS-evoked changes in cue-induced functional connectivity for multiple classes of addicts (e.g., alcohol, nicotine, cocaine, and methamphetamine) and the relationship between functional changes and clinical variables. The current understanding of relationship between dynamic functional changes and selected clinical outcomes is still sparse.

These understandings were addressed in a recent study published on the journal of *Biological Psychiatry: Cognitive Neuroscience and Neuroimaging*, Kearney-Ramos et al. The study utilized two single-blinded, within-subject, and active sham-controlled experiments to investigate whether a single session of 3,600 pulses, 110% resting MT of continuous theta burst stimulation (cTBS) over left VMPFC could change the cue-related FC, which was examined by fMRI scans before and after cTBS (Kearney-Ramos et al., 2018). Previous studies have reported that applying cTBS over VMPFC decreased the associated activity in frontal-limbic network (Hanlon et al., 2016). Kearney-Ramos and colleagues hypothesized that cTBS over VMPFC would restrain cocaine/alcohol cue-associated FC of the left VMPFC and 8 regions of interest (ROIs): ventral striatum, bilateral caudate, bilateral putamen, bilateral insula, and ACC. To test this hypothesis, the present study creatively involved two cohorts (twenty-five cocaine users and 24 alcoholics) in parallel to compare the differences across two drug classes, using the same cTBS protocols and other configurations. Before the cTBS sessions, participants were screened for eligibility, and the demographical data were collected (the Structured Clinical Interview for DSM-IV, Timeline Follow-back, Alcohol Use Disorders Identification Test, Fagerstrom Smoking Inventory, Beck's Depression Inventory and Spielberger State-Trait Anxiety Inventory). Each participant received both real and sham cTBS (one type per visit) and the orders were counterbalanced across participants. Self-report craving was reported before and after each cTBS session. Individual scalp-to-cortex distance was also considered. Results showed that there was significantly decreased FC across eight striatal/salience-VMPFC connections, and the neural activities to cTBS in cocaine users and alcoholics were similar. These findings accorded well with previous studies in cocaine addicts (Hanlon et al., 2015, 2017b), and generalized to alcoholics. Their findings also underscored the importance of relationship between cTBS-evoked brain activities and clinical variables. For cocaine users, it showed positive correlations between FC changes in VMPFC-left caudate and years of cocaine use/baseline craving score. While for alcoholics, FC changes in VMPFC-bilateral caudate/left putamen only correlated with the dose of cTBS, which suggested that there might exist individual variability in the therapeutic response to cTBS. Future studies to ascertain variables (e.g., baseline activity in frontal-limbic network, drug cue reactivity) that could predict rTMS treatment response in drug dependence will be of interest.

Another extensive line of research has demonstrated VMPFC-limbic connections are altered in addicts (Ma et al., 2010). However, triggering some specific neural circuits by TMS in addiction treatment is likely lacking, since brunch of studies focused on the focal stimulation of DLPFC or VMPFC (Nakamura-Palacios et al., 2016; Shen et al., 2016; Liu et al., 2017). This particular research emphasized on function interactions change between the VMPFC-bilateral putamen, VMPFC-bilateral ACC, VMPFC-bilateral insula and VMPF-bilateral caudate after a single cTBS treatment. Nevertheless, a single cTBS treatment failed to reduce the self-reported craving in cocaine and alcohol addicts, neither correlated with the changes in FC. The interaction of frontal-limbic FC recovery and reduced self-reported craving/relapsing rate remain to be tested in the further research.

In conclusion, the current study demonstrated that cTBS of VMPFC-limbic circuits could change drug cue reactivity. Their results are likely to inspire more therapeutic interventions targeting drug-bias attention and self-awareness to modify previously inappropriate rewarded behavior for this chronic repeated relapsing disease. The approach combining fMRI with rTMS will be widely used to understand the underlying pathophysiologic neural circuits, and to design individualized treatment intervention. Nevertheless, although such evidence seems to be informative to us, it cannot effectively reduce drug-induced craving or relieve addiction syndrome. Our previous results indicated more participants respond to five repeated sessions with a better effect on craving in comparison to those who only receive a single session (Shen et al., 2016). To develop the TMS as a promising addiction treatment, the field needs to conduct more research and clinical trials with respect to repeated treatment sessions, optimum treatment protocols (e.g., stimulation parameters, brain target, coil type, duration), long-term follow up and more outcome measures (e.g., withdraw symptoms, relapse symptoms, cognitive scales, impulsivity, sleep).

## Author contributions

All authors listed have made a substantial, direct and intellectual contribution to the work, and approved it for publication.

### Conflict of interest statement

The authors declare that the research was conducted in the absence of any commercial or financial relationships that could be construed as a potential conflict of interest.
